# Effects of Adapted Aquatic Exercise on Autism-Related Behaviors, Flexibility, and Handgrip Strength in Boys with Autism Spectrum Disorder: A Randomized Controlled Trial

**DOI:** 10.3390/healthcare14131838

**Published:** 2026-06-24

**Authors:** Çalık Veli Koçak, Murat Ergin, Can Koçak, Mehmet Savaş Nebol, Mustafa Kayıhan Erbaş, Umut Canlı, Monira I. Aldhahi

**Affiliations:** 1 Faculty of Sport Sciences, Aksaray University, Aksaray 68100, Türkiye; velikocak@aksaray.edu.tr (Ç.V.K.); muratergin@aksaray.edu.tr (M.E.); nebolsavas@gmail.com (M.S.N.); kayihan@aksaray.edu.tr (M.K.E.); 2 Institute of Social Sciences, Aksaray University, Aksaray 68100, Türkiye; cancankocak@gmail.com; 3 Faculty of Sport Sciences, Tekirdağ Namık Kemal University, Tekirdağ 59030, Türkiye; ucanli@nku.edu.tr; 4 Department of Rehabilitation Sciences, College of Health and Rehabilitation Sciences, Princess Nourah bint Abdulrahman University, P.O. Box 84428, Riyadh 11671, Saudi Arabia

**Keywords:** autism spectrum disorder, adapted aquatic exercise, autism-related behaviors, flexibility, handgrip strength

## Abstract

**Background/Objectives**: Autism spectrum disorder (ASD) is a neurodevelopmental condition characterized by impairments in social communication and the presence of restricted and repetitive behaviors, often accompanied by motor impairments. Previous research indicates that regular physical exercise may reduce autism-related behaviors and improve motor competence. This study aimed to examine the effects of an adapted aquatic exercise program on autism-related behaviors, flexibility, and handgrip strength, key motor functions relevant to daily functioning. **Methods**: In this parallel-group randomized controlled trial, 35 boys with mild autism spectrum disorder (aged 8.4 ± 2.1 years) were enrolled. Participants were randomly assigned to an exercise group (*n* = 17) and a control group (*n* = 18). The exercise group completed a 16-week adapted aquatic exercise program (2 sessions/week, 50 min/session), while the control group received usual education only. The primary outcome was autism-related behaviors assessed by the Autism Behavior Checklist (ABC); secondary outcomes included flexibility and handgrip strength. **Results**: The exercise group showed significant improvements in Autism Behavior Checklist (ABC) scores, flexibility, and handgrip strength compared with the control group (*p* < 0.05). Large effect sizes were observed across all outcomes (partial eta squared, ηp^2^ > 0.14). These findings indicate that adapted aquatic exercise confers beneficial effects on behavioral and motor outcomes in children with mild ASD. **Conclusions**: Regular participation in adapted aquatic exercise reduces autism-related behaviors and improves flexibility and handgrip strength. These findings provide empirical support for the inclusion of aquatic exercise in intervention programs targeting children with ASD and may inform future research and practice.

## 1. Introduction

Autism Spectrum Disorder (ASD) is a neurodevelopmental condition characterized by deficits in social behavior and the presence of repetitive behavioral patterns [1]. The global prevalence of ASD is estimated to be approximately 61.8 million people in 2021 (95% uncertainty range: 52.1–72.7 million), which corresponds to approximately 1 in 127 people worldwide [2]. Furthermore, according to the latest data from the Centers for Disease Control and Prevention’s Autism and Developmental Disability Surveillance (ADDM) Network, approximately 1 in 31 (3.2%) 8-year-old children have been diagnosed with ASD in the United States [3].

Although individual characteristics vary, children with ASD commonly exhibit speech impairments, cognitive difficulties, sensory processing problems, repetitive behaviors, and deficits in social and self-care skills [4,5]. Behaviors such as stereotyped movements, lack of responsiveness to environmental stimuli, prolonged staring, rigid adherence to routines, tantrums, absence of speech, or constant echolalia are frequently associated with ASD [6]. Research also indicates that children with ASD are more sedentary than their typically developing peers, which increases their risk of obesity [7,8,9].

Motor impairments are frequently observed in individuals with ASD, although they are not included in the core diagnostic criteria. These impairments may include reduced muscle strength, delayed reaction time, atypical muscle tone, poor coordination, and deficits in postural control [10,11,12,13]. Among motor skills, flexibility and handgrip strength are particularly affected and are critical for independent functioning in daily life [14,15]. Hand grip strength is widely accepted as a valid indicator of overall muscle strength and can be used as a practical, rapid, and reliable measure to assess general muscle function [16]. In addition to reflecting overall muscle capacity, grip strength plays a fundamental role in the performance of daily living activities, including tasks such as eating, brushing teeth, object manipulation, handwriting, and play.

Flexibility represents another critical component of the musculoskeletal system and contributes to efficient movement patterns, postural stability, gait performance, and injury prevention [17]. Given the importance of both muscle strength and flexibility for functional independence and motor performance, their assessment provides valuable insights into physical functioning. Furthermore, compared to comprehensive motor assessment batteries, grip strength and flexibility measurements require minimal instruction, shorter application times, and lower cognitive demands. Therefore, it offers a more practical and potentially more reliable approach to assessing physical fitness in children with ASD [16,17].

One evidence-based approach to developing these skills is structured physical exercise, and aquatic exercises are among the recommended methods [18,19]. Aquatic exercises provide physical gains through turbulence and resistance: the buoyancy-resistance interaction requires individuals to apply force to stay afloat, which increases muscle demand compared to work done on land. The turbulent and inherently unpredictable nature of the aquatic environment necessitates constant posture adjustments and supports the development of balance, coordination, and motor control [20]. In addition, hydrostatic pressure increases respiratory load and can increase lung capacity by up to 60% [21].

In addition to these physical parameters, the aquatic environment is considered to provide multiple sensory stimulation through thermal structure, low gravity, and vestibular system inputs. Indeed, the act of swimming supports the sensory integration process at a therapeutic level by providing both tactile and kinesthetic inputs to the central nervous system [22]. Beyond these physiological and neurological effects, the social interaction and play opportunities offered by water activities facilitate language acquisition processes and optimize psychosocial components such as self-esteem, self-awareness, and a sense of accomplishment [23]; furthermore, children with ASD have a 160 times higher risk of drowning than the general population, making aquatic exercises critically important in this respect [24]. Families of children with ASD frequently report heightened concern and negative impact on family life due to drowning risk [25].

Thus, aquatic exercises provide multidimensional support for the development of children with ASD. Indeed, although limited in number, studies have shown that aquatic exercise improves motor, social, and swimming skills in this population [26,27,28]. For example, Zanobini and Solari [29] found that aquatic exercise significantly reduced autism-related behaviors in children with ASD. Similarly, researchers demonstrated that technique-based and play-based aquatic programs improved gross motor skills and repetitive behaviors [26]. Pan [30] reported that aquatic exercise improved both flexibility and aquatic skills in children with ASD and their typically developing siblings.

Evidence supporting water-based interventions for individuals with AS) is increasing. However, to current information, only six studies have specifically focused on autism-related behaviors, and only four of these included a control group. Studies that assess both motor skills and autism-related behaviors within an experimental framework and use blinded methods are limited. Furthermore, a review of the literature reveals that some studies include heterogeneous participant samples or address behavioral and motor outcomes separately. For example, in the study conducted by Fragala-Pinkham et al. [31], only three participants were diagnosed with ASD, while the remaining participants had other neurodevelopmental disorders.

On the other hand, when current research involving only individuals with ASD is examined, it is seen that the number of studies addressing autism-related behavioral outcomes along with basic physical fitness indicators such as flexibility and muscle strength is limited. Previous research has primarily focused on specific areas such as stereotypical behaviors [26], autism-specific behaviors [32], or the behavioral effects of water activities [29]. While these studies have provided valuable information about the behavioral benefits of aquatic exercise, they have not assessed behavioral symptoms simultaneously with flexibility and strength outcomes.

In this context, the literature on the combined assessment of behavioral and physical fitness changes following aquatic exercise interventions in children with ASD appears limited. Adopting a multidimensional assessment framework encompassing both behavioral and physical outcomes, this study aims to provide more comprehensive evidence on the potential benefits of aquatic exercise for children with ASD. It is believed that this methodological approach can contribute to the existing literature and support the development of evidence-based intervention strategies for this population.

Research Question 1:

Does participation in an adapted aquatic exercise reduce autism-related behaviors in pre-adolescent children with mild ASD?

Research Question 2:

Does participation in an adapted aquatic exercise improve flexibility and handgrip strength in pre-adolescent children with mild (ASD)?

Based on existing evidence and the aims of this study, the following hypotheses were formulated:

H^1^: Adapted aquatic exercise reduces autism-related behaviors in pre-adolescent children with mild ASD.

H^2^: Adapted aquatic exercise improves flexibility and handgrip strength in pre-adolescent children with mild ASD.

## 2. Materials and Methods

### 2.1. Research Design

**Figure 1:**
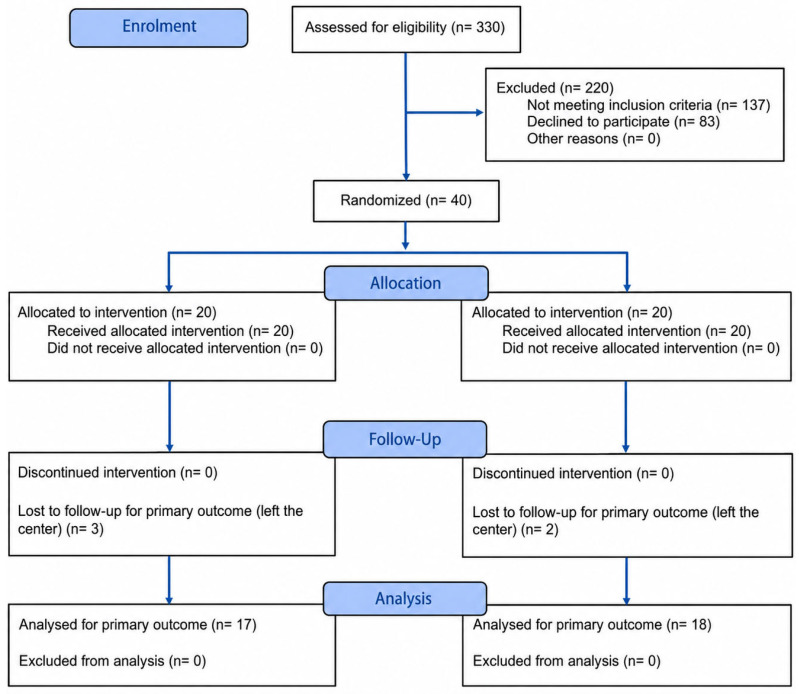
CONSORT flow chart.

This study adhered to the CONSORT (Figure 1) guidelines [33]. The study protocol was prospectively registered on ClinicalTrials.gov under the identifier NCT07410897. A parallel-group, randomized controlled experimental design with pre-test and post-test measurements was employed.

### 2.2. Sample Size Estimation and Participants

The sample size was estimated using G*Power software (version 3.1.9.7; Heinrich Heine University Düsseldorf, Düsseldorf, Germany) [34] for a repeated measures analysis of variance (ANOVA) design involving two groups (exercise and control) and two time points (pre-test and post-test). The required sample size was calculated assuming a moderate effect size (Cohen’s f = 0.25), a two-way significance level of α = 0.05, and statistical power (1 − β) = 0.80. The correlation between repeated measures was determined as r = 0.50, and the sphericity assumption was met (ε = 1). The analysis showed that a total of 34 participants would be sufficient to detect a significant group × time interaction effect. Participants were drawn from a pool of 330 students diagnosed with ASD. Of these, 110 were identified as having mild ASD. Forty participants who met the inclusion criteria and agreed to participate in the study were randomly assigned to either the exercise group (*n* = 20) or the control group (*n* = 20) (Figure 1).

Participants were male children diagnosed with ASD and enrolled at the SOBE Foundation (Selçuklu Foundation for the Education of Individuals with Autism), Türkiye. Throughout the 2025 academic year, the foundation provided a variety of intervention services, including educational support, hydrotherapy, movement training, occupational therapy, and hippotherapy. Participants received one or more of these services according to their individual needs and intervention plans. All participants had a confirmed clinical diagnosis of mild ASD established (Level 1) by a child psychiatrist in accordance with the diagnostic criteria of the Diagnostic and Statistical Manual of Autistic Disorders DSM-5 criteria [35].

Inclusion criteria were as follows: (a) a clinical diagnosis of mild ASD, (b) medical clearance for participation in aquatic exercise, (c) ability to follow verbal and visual instructions, (d) ability to imitate fine and gross motor movements, (e) absence of orthopedic disability, (f) no involvement in structured sports programs other than academic instruction and the adapted aquatic exercise program, (g) independence in toilet, and (h) written informed consent provided by parents or legal guardians.

Three participants from the EG and two from the CG withdrew the study for personal reasons before the intervention was completed. Thus, 35 participants (EG = 17; CG = 18) completed the study. Only data from participants who successfully completed the intervention were analyzed.

### 2.3. Adapted Aquatic Exercise Program

Both the adapted aquatic exercise program and standard academic instruction were implemented over a 16-week period. The intervention duration was determined based on previous studies indicating that aquatic exercise programs lasting between 8 and 16 weeks are sufficient to produce meaningful behavioral and motor outcomes in children with ASD [27,29,36].

The adapted aquatic exercise program was jointly developed by a certified adapted swimming coach and a special education specialist. Program objectives focused on water safety and acclimatization, breath control, lower and upper limb propulsion, and the acquisition of fundamental freestyle swimming skills. To ensure consistency in intervention implementation, a 30-min briefing session was held for all instructors prior to implementation. During the briefing session, instructors reviewed the intervention protocol, weekly goals, training procedures, participant progress criteria, and safety procedures. Prior to implementation, instructors reviewed the intervention protocol and weekly training goals. Furthermore, all instructors followed the same predefined intervention framework and training goals throughout the study.

The intervention consisted of 32 scheduled sessions (two 50-min sessions per week) over 16 weeks, corresponding to a total scheduled training volume of 1600 min. Attendance at all exercise sessions in the experimental group was recorded, and participants completed 87% of the planned intervention sessions. This corresponds to an average training volume of approximately 1392 min. Participants who missed scheduled sessions were offered make-up sessions whenever possible to maximize adherence to the intervention protocol.

All intervention sessions were conducted by certified adapted swimming instructors with lifeguard certifications. All sessions were one-on-one, and progress toward training goals was based on individual skill mastery, not a fixed schedule. Consequently, minor adjustments were made to weekly goals depending on each participant’s learning pace. For example, the lowest-performing participant completed the program at training levels 7–8, corresponding to the “freestyle arm and leg movements” phase.

Exercise intensity was monitored using the OMNI Scale of Perceived Effort for Children, with a target range of 5–7 representing moderate intensity. Sessions were scheduled between 09:00 and 16:20 on weekdays, depending on instructor, participant, and pool availability. Sessions were held on non-consecutive days. All sessions were conducted in an indoor pool 10 m long and 1–1.5 m deep. The detailed weekly structure and training objectives of the adapted aquatic exercise program are presented in Table 1.

### 2.4. Experimental Procedure

Outcome measures included the Autism Behavior Checklist (ABC) to assess autism-related behaviors as well at the sit and reach and handgrip strength tests for evaluate selected motor skills. Data collection was conducted at two time points, separated by a 16-week intervention period. At baseline, demographic information (age, body mass index, autism severity, etc.), ABC scores, sit and reach flexibility, and handgrip strength measurements were obtained. Following baseline assessment, the exercise group participated in the adapted aquatic exercise program in addition to their regular academic instruction, whereas the control group continued to receive academic instruction only.

Post-intervention assessments were conducted by the same assessment team using identical measurement instruments and procedures, following the same sequence as at baseline. All assessments were supervised by certified psychological counseling and guidance teachers. Measurements for both groups were administered individually in the same gymnasium setting, with care taken to ensure that participants did not observe one another during testing.

Several measures were taken in the study to minimize bias. Water exercise instructors followed a standardized intervention protocol and were not included in the pre-test and post-test flexibility and handgrip tests and data analysis. Furthermore, evaluators of objective motor outcomes (flexibility and handgrip strength) were blinded to the group assignment. However, teachers who completed the Autism Behavior Checklist (ABC) were aware of the participants’ group assignments.

Data analysts were blinded to group assignment. Since the intervention involved exercise sessions, blinding both participants and researchers was not possible. However, an externally blinded researcher performed the randomization and delivered the group assignments to the first researcher in sealed envelopes. Pre-test and post-test data were collected and analyzed by the blinded researcher.

All analyses were conducted using the complete available dataset. Pre-test and post-test measurements were obtained from all participants, and no missing data were identified; therefore, all cases were included in the final statistical analyses.

### 2.5. Autism Behavior Checklist (ABC)

The Autism Behavior Checklist (ABC) was developed by Krug et al. [37] as a screening tool for ASD and as a measure of the severity of autism-related behaviors. The Turkish adaptation demonstrated strong validity and reliability (α = 0.92) [38].

The checklist consists of 57 items across five subdomains: Sensory (9 items), Relating (12 items), Body and Object Use (12 items), Language Skills (13 items), and Social and Self-Help Skills (11 items). Items are weighted on a scale from 1 to 4 based on symptom severity, with higher total scores indicating greater levels of autism-related behaviors. In the present study, the ABC was completed by the participants’ classroom teachers, who were familiar with the children’s daily behavioral patterns.

### 2.6. Sit and Reach Test

The sit and reach test evaluates posterior chain flexibility and has been shown to be reliable for children with ASD [39]. A standardized sit and reach box (01285A, Lafayette Instrument Company, Lafayette, IN, USA; accuracy 0.1 cm) was used. Participants sat barefoot with feet placed against the box, reaching forward with both hands aligned. Three trials were administered, and the best score (cm) was recorded [40,41].

### 2.7. Handgrip Strength Test

Handgrip strength was assessed using a Takei dynamometer (TKK 5101, Takei Scientific Instruments Co., Ltd., Tokyo, Japan; accuracy 0.100 kg). This protocol is reliable for individuals with ASD [39]. The handle was adjusted to hand size, and participants squeezed maximally while standing in anatomical position. Two trials were administered, with a third added if differences exceeded 10% [42].

### 2.8. Statistical Analysis

Statistical analyses were performed using SPSS software (Version 27, IBM, Chicago, IL, USA). The significance level was set at *p* < 0.05. Assumptions of normality (Shapiro–Wilk test) and homogeneity of variance (Levene’s test) were evaluated. Descriptive statistics were presented as means and standard deviations. The reliability of the outcome measures (ABC, handgrip strength, and flexibility) was evaluated using the intraclass correlation coefficient (ICC), based on a two-way random-effects model assessing absolute agreement for single measurements [ICC (2,1)]. ICC values were calculated separately for each test with 95% confidence intervals to ensure measurement stability. The reliability analysis demonstrated good to excellent measurement consistency across all outcomes. According to established interpretation guidelines, ICC values above 0.75 indicate good reliability, while values above 0.90 indicate excellent reliability [43], supporting the robustness of the measurement procedures used in this study. A 2 × 2 repeated-measures ANOVA (group × time) was used to examine changes over time and between groups. Box’s M test was used to assess homogeneity of covariance matrices. The assumption of Sphericity was tested using Mauchly’s test. When the sphericity assumption was violated, the Greenhouse–Geisser correction was applied. Effect sizes were calculated using partial eta squared (ηp^2^), which indicates the proportion of variance in the dependent variable explained by the independent variable. According to, ηp^2^ values small (0.01–0.06), medium (0.06–0.14), or large (>0.14) [44,45].

## 3. Results

This section presents the results of the statistical analyses conducted to evaluate the effects of an adapted aquatic exercise program on autism-related behaviors, flexibility, hand grip strength, and anthropometric characteristics.

According to Table 2, the participants had a mean age of 8.4 years (±2.1), a mean height of 131.66 cm (±17.15), a mean body mass of 31.71 kg (±12.67) and a mean body mass index (BMI) of 17.58 kg/m^2^ (±3.28). No significant group × time interaction effects were observed for height (F(1,33) = 2.574, *p* = 0.118, ηp^2^ = 0.072), body weight (F(1,33) = 0.001, *p* = 0.980, ηp^2^ = 0.001), or body mass index (BMI) (F(1,33) = 0.061, *p* = 0.810, ηp^2^ = 0.002). Repeated-measures ANOVA revealed no significant group × time interaction effects for height, body weight, or BMI (*p* > 0.05). These findings indicate that changes in anthropometric characteristics over the 16-week intervention period did not differ significantly between the exercise and control groups.

Table 3 presents the raw descriptive statistics (mean ± standard deviation) of ABC scores, flexibility, and handgrip strength for the exercise and control groups, along with within-group and between-group comparisons.

The analysis of test–retest reliability showed high intraclass correlation coefficients (ICC) for all outcome measures. Intraclass correlation coefficients (ICC) with 95% confidence intervals were 0.78 (95% CI: 0.62–0.88) for ABC, 0.76 (95% CI: 0.58–0.87) for handgrip strength, and 0.92 (95% CI: 0.84–0.97) for flexibility, indicating stable measurement properties across repeated assessments. A 2 × 2 mixed-design repeated-measures ANOVA revealed significant group × time interaction effects for all outcomes.

Autism Behavior Checklist (ABC) scores decreased significantly in the exercise group from 47.94 ± 16.89 (95% CI: 37.36–58.52) at pre-test to 27.59 ± 9.39 (95% CI: 17.60–37.57) at post-test, whereas the control group remained largely unchanged (61.77 ± 24.97 [95% CI: 51.50–72.06] vs. 61.11 ± 26.67 [95% CI: 51.41–70.81]); F(1,33) = 10.211, *p* = 0.003, ηp^2^ = 0.24.

Flexibility improved in the exercise group (14.94 ± 8.51 cm [95% CI: 12.07–17.84] to 18.00 ± 9.48 cm [95% CI: 13.51–22.49]), while the control group showed a slight decrease (13.78 ± 7.88 cm [95% CI: 9.85–17.71] to 13.11 ± 8.74 cm [95% CI: 8.74–17.48]); F(1,33) = 6.327, *p* = 0.017, ηp^2^ = 0.16.

Handgrip strength increased markedly in the exercise group (5.75 ± 2.83 kg [95% CI: 4.26–7.23] to 9.60 ± 3.32 kg [95% CI: 7.94–11.27]), with smaller gains in the control group (5.40 ± 3.16 kg [95% CI: 3.96–6.84] to 6.64 ± 3.42 kg [95% CI: 5.02–8.26]); F(1,33) = 8.706, *p* = 0.006, ηp^2^ = 0.21.

These results indicate statistically significant differences in change over time between the exercise and control groups across all outcome measures. When the effect sizes were examined, a large effect size was detected in all parameters where there was a significant difference (ηp^2^ > 0.14), indicating substantial practical significance.

**Figure 2:**
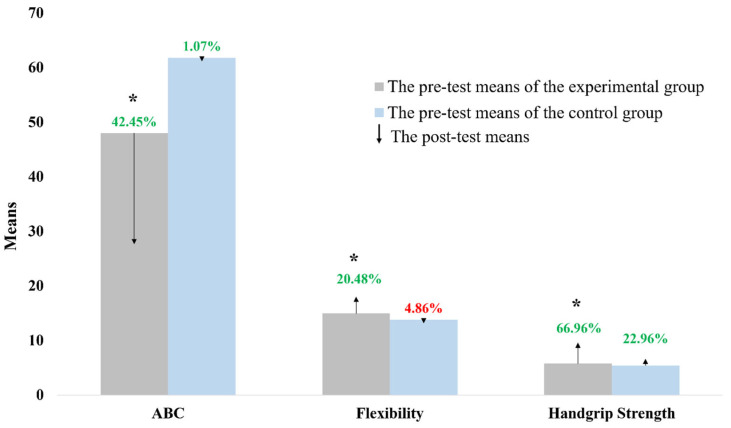
Pre-test, post-test percentage changes in the exercise and control groups.

Figure 2 percentage change in ABC scores, flexibility, and handgrip strength from pre-test to post-test in the exercise and control groups. Percentage changes were calculated relative to baseline (pre-test) values and are presented for descriptive purposes to illustrate the magnitude and direction of change between groups. Group differences in change trajectories are consistent with the significant group × time interaction effects identified in the repeated-measures ANOVA.

In Figure 2, arrows indicate the direction of change from the pre-test averages to the post-test averages. The percentages shown in green indicate an increase in performance, whilst those shown in red indicate a decrease in performance. The * symbol indicates that there is a statistically significant difference between the groups and shows in favour of which group this difference lies (*p* < 0.05).

In the exercise group, ABC scores decreased by 42.45%, while the control group showed a minimal change of 1.07%. Flexibility increased by 20.48% in the exercise group compared with a 4.86% decrease in the control group. Handgrip strength increased by 66.96% in the exercise group and 22.96% in the control group.

## 4. Discussion

### 4.1. Interpretation of Main Findings

In this study, no significant group × time interaction was found for height, body weight, or BMI following a 16-week adapted aquatic exercise program. These findings suggest that participation in the aquatic exercise program did not affect anthropometric variables related to growth beyond what would be expected from normal development. Increases in height and body weight in participants in both groups may reflect normal growth trajectories rather than the effects of the aquatic exercise intervention. The primary goals of the adapted aquatic exercise program focused on improving aquatic adaptability skills and swimming skills. Previous research has shown that aquatic exercise interventions in children with ASD primarily improve motor skills, aquatic skills, social interaction, and behavioral outcomes, while changes in body weight and BMI are generally less significant [36,46].

This present study examined the effects of an adapted aquatic exercise program implemented alongside academic instruction on autism-related behaviors, flexibility, and handgrip strength in male children with ASD. The findings indicate that regular participation in adapted aquatic exercises significant reductions in autism-related behaviors and meaningful improvements in both flexibility and handgrip strength.

The observed reduction in autism-related behaviors is consistent with previous research examining aquatic interventions for children with ASD. Caputo, Ippolito, Mazzotta, Sentenza, Muzio, Salzano and Conson [32], who implemented a 10-month aquatic program, reported substantial decreases in autism-related behaviors alongside improvements in swimming competence. Similarly, Zanobini and Solari [29], demonstrated that a 20-week aquatic intervention resulted in significant behavioral improvements that were maintained during a six month follow-up period. These findings suggest that aquatic exercise programs of sufficient duration may yield stable behavioral benefits.

Marzouki, Soussi, Selmi, Hajji, Marsigliante, Bouhlel, Muscella, Weiss and Knetchtle [26], using a control-group experimental design, compared technique-based and play-based aquatic programs delivered over eight weeks. Although no differences were found between the two exercise modalities, both exercise groups demonstrated significantly greater improvements in gross motor skills and reductions in repetitive behaviors compared with the control group.

Although the number of studies directly examining the effects of aquatic exercise on autism-related behaviors remains limited, autism is fundamentally characterized by deficits in social functioning [46]. Therefore, evaluating the present findings in the context of aquatic-exercise research on social skills provides meaningful insight. For example, Pan [27] reported that a 10-week aquatic program improved both aquatic skills but also social behaviors in boys with ASD. Similarly, Chu and Pan [36] reported that a 16-week peer and sibling assisted aquatic program significantly enhanced social behaviors in children with ASD. Battaglia et al. [47] extended these findings to adolescents with ASD, showing significant improvements in social behavior following an aquatic program adapted from Caputo and colleagues’ model.

Additional evidence comes from Mills et al. [48], who employed a randomized crossover design and observed marked gains in social skills and well-being following a four-week hydrotherapy intervention. Likewise, Güeita-Rodriguez, Ogonowska-Slodownik, Morgulec-Adamowicz, Martin-Prades, Cuenca-Zaldivar and Palacios-Cena [18], using a mixed-methods approach, reported consistent improvements in the social behaviors based on both parental reports and quantitative measures after a seven-month adapted aquatic program. In contrast, Alaniz et al. [49] reported improvements in aquatic skills at multiple assessment points but were unable to evaluate social behaviors beyond eight week due to methodological constraints.

Taken together, the present findings and existing literature suggest that adapted aquatic exercise may effectively reduce autism-related behaviors and enhance social functioning in children with ASD. Potential mechanisms underlying these effects include improved body awareness, regulation of physiological arousal, reduction in anxiety and stress, and increased perceptions of competence and success within a supportive and structured environment.

In addition to behavioral outcomes, the present study demonstrated significant improvements in flexibility and handgrip strength. These results align with Yılmaz, Yanardağ, Birkan and Bumin [28], who reported significant improvements in flexibility and handgrip strength in a child with ASD following a 10-week aquatic intervention, accompanied by reductions in repetitive behaviors. Similarly, Pan [30] observed improvements in flexibility and aquatic skills in both children with ASD and their typically developing siblings following a 14-week aquatic program. Although limited in number, other studies have also reported enhancements in swimming skills and other motor abilities following aquatic interventions in children with ASD [31,50,51].

However, it should not be overlooked that feedback, motivational support, and individualized guidance provided by instructors during the intervention process in the current study may be among the factors influencing participants’ level of engagement and performance in the program. Nevertheless, psychosocial processes such as the quality and frequency of instructor–participant interaction were not systematically evaluated within the scope of this study. Therefore, it is not possible to definitively determine the extent to which the observed improvements are directly attributable to the aquatic exercise intervention and the extent to which they are attributable to the educational and motivational interactions within the intervention process. Future research evaluating these psychosocial variables could contribute to a more comprehensive understanding of the mechanisms underlying the intervention effects.

It is assessed that improvements in participants’ swimming technique and aquatic movement skills during the intervention process may have influenced performance outcomes by increasing movement economy and improving exercise tolerance. Therefore, the observed improvements may not be explained solely by training-related physiological adaptations; motor learning processes and technical skill development may also have contributed to these results.

The observed motor improvements may be attributed to the unique physical properties of aquatic exercise. Increases in handgrip strength may stem from repeated hand-based force production required for flotation, propulsion, and postural stabilization. Improvements in flexibility may reflect the engagement of multiple muscle groups and the resistance characteristics of water, which promote greater movement amplitude and joint mobility while reducing joint loading. In contrast, the decline in flexibility observed in the control group highlights the importance of structured physical activity during periods of growth and development.

Overall, the findings of this study support the therapeutic potential of adapted aquatic exercise for improving both behavioral and motor outcomes in children with ASD.

### 4.2. Recommendations

When the findings of the current study and the evidence in the relevant literature are considered together, it is thought that adapted aquatic exercise may provide potential benefits in reducing behavioral symptoms and supporting motor performance in children with ASD. Accordingly, integrating structured aquatic exercise programs into education, rehabilitation, and recreation-based services can be considered a complementary element of multi-component intervention approaches for children with ASD.

However, the feasibility and sustainability of such programs can be affected by various structural and organizational factors such as the availability of appropriate physical infrastructure, safe aquatic environments, trained instructors, and sufficient support staff. Therefore, it is important to consider these conditions in the planning and dissemination processes of intervention programs. Furthermore, it is anticipated that interdisciplinary and inter-institutional collaborations between educational institutions, rehabilitation centers, local governments, and community based recreation organizations can increase the accessibility of aquatic exercise programs and support their long term applicability.

Future research should include studies with larger samples encompassing individuals with ASD across different age groups and functional levels, as well as research designs that allow for long-term follow-up of intervention effects. Such studies are expected to contribute to strengthening the current evidence base regarding the effects of aquatic exercise on behavioral and physical outcomes. Furthermore, a multi-source assessment framework, including parent-reported measures and blinded independent observations, should be adopted to enhance the validity and reliability of outcome assessment.

Finally, future studies should not be limited to behavioral scales such as the ABC, but should also include standardized measures of daily living skills and adaptive behavior, allowing for a more comprehensive assessment of the intervention’s impact on real life functioning.

### 4.3. Strengths of the Study

This study achieved a high attendance rate (87%), which reached 100% when make-up sessions were included. The 16-week intervention period exceeded the duration commonly reported in similar studies. Moreover, by simultaneously evaluating behavioral and motor outcomes within a controlled design, the study contributes uniquely to the limited literature. Notably, this research is the first to specifically examine handgrip strength and flexibility two motor skills with high relevance for daily functioning within an adapted aquatic intervention for children with ASD.

### 4.4. Limitations

The current research possibilities should be carefully evaluated in terms of potential outcomes for wider applications. Although the results are promising, long-term follow-up data are needed from larger sample groups, multicenter, independent, repeated studies of adapted water exercise in different regions and diverse application settings.

The main limitations of the study are that behavioral outcomes were obtained solely based on teacher reports, and the study was conducted as a single-center study. The teachers who completed the ABC assessments were aware of the group assignments, and this is one of the methodological limitations of the study. Specifically, we acknowledge that the failure to blind the teachers may have led to potential bias. Furthermore, behavioral outcomes were derived exclusively from teacher reports; therefore, the absence of data triangulation from alternative informants, such as parents or independent observers, may limit the robustness and generalizability of the findings. Another limitation of the study is that it only included boys with mild ASD. Therefore, the generalizability of the findings to boys with moderate and severe ASD is limited, and future studies should evaluate the effectiveness of adapted water exercise programs at different levels of ASD severity. In addition, the inability to control for variables related to the child’s socioeconomic status and the parent’s education level prevented the evaluation of the potential impacts of this individual intervention on outcomes.

## 5. Conclusions

This study examined the effects of an adapted aquatic exercise program, implemented in addition to academic instruction, on autism-related behaviors, flexibility, and handgrip strength in preadolescent children with ASD. Results from this parallel-group randomized controlled trial, supported by existing literature, indicate that regular participation in adapted aquatic exercise effectively reduces autism-related behaviors while improving flexibility and handgrip strength. No adverse effects were reported by parents or teachers throughout the intervention. Overall, these results are consistent with limited research examining aquatic exercise in children with ASD, and adapted aquatic exercise may be a promising intervention that can serve as a therapeutic method to improve both behavioral and motor functions in this population.

## Figures and Tables

**Table 1:** Content of the adapted aquatic exercise program.

Week	Goals	Events
1–2	Pool Rules and Getting to Know the Water	Introducing swimming equipment and supplies. Teaching pool entry and exit rules. Removing undesirable behaviors.
3–4	Getting Used to Water, Breathing Exercises	Entering the water independently with flotation devices. Engaging in aquatic games. Practice gliding without technique in the water.
5–6	Getting Used to Water, Breathing Exercises	Performing blowing exercises on land and in the pool. Dipping your head into the water. Playing with fetch toys underwater.
7–8	Freestyle Arm and Footwork	Performing footwork on land. Performing footwork with the body partially immersed in the pool. Performing footwork with your whole body in the pool, holding on to the edge. Performing footwork with a kickboard.
9–10	Freestyle Arm and Footwork	Sliding in the water by pushing off from the edge of the pool and kicking with protective equipment.
11–12	Freestyle Arm Pull Exercise	Performing arm pulls on land. Performing strokes holding on to the edge of the pool. Performing strokes wearing fins and pull-ups.
13–14	Arm and Breath Coordination Exercises	Performing sideways breathing exercises on land. Performing sideways breathing exercises while holding on to the edge of the pool. Performing three-arm, one-breath cycle exercises with fins and kickboard.
15–16	Freestyle Swimming	Three-arm, one-breath cycle freestyle swimming without any supporting equipment. short- and long-distance swimming to improve physical fitness (purposeful training).

**Table 2:** Summary of ANOVA results of participants’ anthropometric measurements.

Variables	EG (*n* = 17)		CG (*n* = 18)		ANOVA		
					Interaction (Group × Time)		
	Pre-Test	Post-Test	Pre-Test	Post-Test			
	M (SD) (95% CI)	M (SD) (95% CI)	M (SD) (95% CI)	M (SD) (95% CI)	F	*p*	ηp^2^
**Height** **(cm)**	137.47 (18.62) 129.38 to 145.56	139.24 (17.83) 131.32 to 147.15	126.17 (14.00) 118.30 to 134.03	129.17 (14.14) 121.48 to 136.86	2.574	0.118	0.072
**Weight** **(kg)**	35.83 (14.83) 29.82 to 41.84	37.87 (15.26) 31.50 to 44.23	27.83 (9.00) 21.98 to 33.67	29.89 (10.20) 23.70 to 36.08	0.001	0.98	0.001
**BMI** **(score)**	18.16 (3.67) 16.54 to 19.78	18.77 (4.03) 16.82 to 20.72	17.03 (2.87) 15.47 to 18.60	17.52 (3.89) 15.62 to 19.42	0.061	0.81	0.002

**Table 3:** Summary of ANOVA results of participant performances.

Variables	EG (*n* = 17)		CG (*n* = 18)		ANOVA		
					Interaction (Group × Time)		
	Pre-Test	Post-Test	Pre-Test	Post-Test			
	M (SD) (95% CI)	M (SD) (95% CI)	M (SD) (95% CI)	M (SD) (95% CI)	F	*p*	ηp^2^
**ABC (score)**	47.94 (16.89) (37.36 to 58.52)	27.59 (9.39) (17.60 to 37.57)	61.77 (24.97) (51.50 to 72.06)	61.11 (26.67) (51.41 to 70.81)	10.211	0.003 *	0.24
**Flexibility (cm)**	14.94 (8.51) (10.90 to 18.98)	18.00 (9.48) (13.51 to 22.49)	13.78 (7.88) (9.86 to 17.71)	13.11 (8.74) (8.74 to 17.48)	6.327	0.017	0.16
**Handgrip Strength** **(kg)**	5.75 (2.83) (4.26 to 7.23)	9.60 (3.32) (7.94 to 11.27)	5.40 (3.16) (3.96 to 6.84)	6.64 (3.42) (5.02 to 8.26)	8.706	0.006 *	0.21

**Note:** ABC = The Autism Behavior Checklist; EG = exercise group; CG = control group; * *p* < 0.05.

## Data Availability

The data presented in this study are available from the corresponding author upon request due to participant confidentiality.

## Abbreviations

The following abbreviations are used in this manuscript:

ASD	Autism spectrum disorder
EG	Exercise group
CG	Control group
